# Supplementary material for: Chemical Interpretation of Time-Dependent Coupled-Cluster Theory
Source: J Phys Chem A. 2026 Aug 3;130(32):6475–95. doi: 10.1021/acs.jpca.6c03228 (PMC13479612; doi:10.1021/acs.jpca.6c03228)
Supplement: Supplementary file 1 [file jp6c03228_si_001.pdf]

Supporting Information for "Chemical Interpretation of Time-Dependent Coupled-Cluster Theory"

Aparna Krishnan,[†] Håkon Emil Kristiansen,[‡] Benjamin G. Peyton,[¶] T. Daniel Crawford,[*,†] and Thomas Bondo Pedersen[*,‡]

†*Department of Chemistry, Virginia Tech, Blacksburg, VA 24061, USA*

‡*Hylleraas Centre for Quantum Molecular Sciences, Department of Chemistry, University of Oslo, P.O. Box 1033 Blindern, N-0315 Oslo, Norway*

¶*Department of Chemistry, Michigan State University, East Lansing, MI 48824, USA*

E-mail: crawdad@vt.edu; t.b.pedersen@kjemi.uio.no

Table S1: HF (a.u.)

Atom Type	X	Y	Z
H	0.000000000000	0.000000000000	-1.645584717549
F	0.000000000000	0.000000000000	0.087294782451

Table S2: H_2O (a.u.)

Atom Type	X	Y	Z
O	0.000000000000	0.000000000000	-0.123909362479
H	-0.000000000000	1.429937284000	0.983265750521
H	0.000000000000	-1.429937284000	0.983265750521

Table S3: NH_3 (a.u.)

Atom Type	X	Y	Z
N	0.000000000000	-0.000005919139	0.118974684932
H	0.000000000000	1.764094080861	-0.551025315068
H	1.527700000000	-0.882005919139	-0.551025315068
H	-1.527700000000	-0.882005919139	-0.551025315068

Table S4: CH_4 (a.u.)

Atom Type	X	Y	Z
C	0.000000000000	0.000000000000	0.000000000000
H	1.697763381629	0.000000000000	1.200500000000
H	-1.697763381629	-0.000000000000	1.200500000000
H	0.000000000000	1.697763381629	-1.200500000000
H	0.000000000000	-1.697763381629	-1.200500000000
